# Brain microstructural damage through serial diffusion tensor imaging and outcomes in Susac syndrome: A prospective cohort study

**DOI:** 10.1111/ene.70002

**Published:** 2024-12-16

**Authors:** Augustin Gaudemer, Marie‐Cécile Henry‐Feugeas, Marion Peyre, Alexandra Kachaner, Isabelle Klein, Antoine Khalil, Thomas Papo, Karim Sacré, Olivier Aumaitre, Anne Catherine Bachoud Levi, Naima Beldjoudi, Marie Bodenant, Jean Capron, Marie‐Paule Chauveheid, Fleur Cohen Aubart, Clémence David, Thomas de Brouker, Solene De Gaalon, Sabrina Debruxelles, Serge Doan, Antoine Dossier, Emmanuel Ellie, Chrystelle François, Sophie Godard‐Ducceschi, Bertrand Godeau, Tiphaine Goulenok, Deborah Grosset‐Janin, Caroline Halimi, Mohamed Hamidou, Eric Jouvent, Cedric Laouenan, Bertand Lapergue, Alain Le Quellec, Arthur Mageau, Elisabeth Medeiros de Bustos, Shirine Mohamed, Luc Mouthon, Jean‐Baptiste Noury, Diane Rouzaud, Mélanie Roriz, Guillaume Turc, Anne Wacongne

**Affiliations:** ^1^ Department of Radiology, Hospital Bichat‐Claude Bernard, Assistance Publique Hôpitaux de Paris Université Paris Cité Paris France; ^2^ Department of Internal Medicine, Hospital Bichat‐Claude Bernard, Assistance Publique Hôpitaux de Paris Université Paris Cité Paris France; ^3^ Department of Radiology Clinique Alleray‐Labrouste Paris France; ^4^ Laboratoire d'Excellence Inflamex, Faculté de Médecine site Bichat, Centre de Recherche sur l'Inflammation, INSERM UMR1149, CNRS ERL8252 Paris France

**Keywords:** brain microstructural damage, diffusion tensor imaging, outcome, Susac syndrome

## Abstract

**Background:**

Susac syndrome (SuS) is a rare immune‐mediated microangiopathy with potential disabling evolution. We aimed to analyze brain microstructural damage through diffusion tensor imaging (DTI) in SuS and determine its association with poor outcomes.

**Method:**

CarESS study is a prospective multicenter national cohort study of patients with SuS. Patients included at the principal investigator's center with at least two available brain magnetic resonance imaging (MRI) with DTI were analyzed. Mean diffusivity (MD) and fractional anisotropy (FA) were measured in fibers crossing three regions of interest (ROIs): the corpus callosum as a whole, the genu of the corpus callosum, and the splenium of the corpus callosum. The primary outcome was work resumption.

**Results:**

Twenty‐two patients (36 (25;42) years, 16 (73%) females) were studied. The triad (i.e., brain, eye, and ear involvement) was complete in 21 (95%) patients. All but one patients received steroids alone or in combination with immunosuppressive drugs (*n* = 11) and/or IVIg (*n* = 7). Over a median follow‐up of 6 (5;8) years, 15 (68%) patients went back to work. FA and MD were longitudinally measured in 123 DTI MRI accounting for a median of 5.6 [4.2; 7] MRI per patient. Microstructural damages in the corpus callosum as a whole, the genu of the corpus callosum, and the splenium of the corpus callosum increased during follow‐up and were significantly associated with the inability to return to work.

**Conclusion:**

Brain DTI identified microstructural damage in fibers crossing the corpus callosum that are associated with long‐term disability in SuS.

**Trial Registration:**

ClinicalTrials.gov portal identifier: NCT01481662 (https://clinicaltrials.gov/ct2/show/NCT01481662?term=caress&draw=2&rank=5).

## INTRODUCTION

Susac syndrome (SuS) is a rare immune‐mediated small vessel disease affecting the brain, the retina, and the inner ear [1, 2, 3]. Diagnosis relies on three sets of features including (i) subacute encephalopathy with unusual headache and pseudo‐psychiatric symptoms associated with multifocal ischemic white matter and corpus callosum lesions, (ii) eye involvement with bilateral occlusions of the branches of the central artery of the retina, and (iii) cochleo‐vestibular damage with neurosensorial hearing loss [4]. SuS is a disabling disease that mostly affects young women [5, 6]. Relapses are frequent, and there are no known predictive factors for poor outcomes [7].

Diffusion tensor imaging (DTI) assess neural tracts through qualitative and quantitative parameters including fractional anisotropy (FA) and mean diffusivity (MD). FA and MD reflect the organization of the white mater fibers and identify microstructural brain damage in cerebral small vessel disease [8, 9, 10, 11, 12, 13, 14]. Interestingly, evidence of white mater damage in SuS, particularly in the corpus callosum, has been reported in small series [15, 16, 17].

The aim of this study was to describe, through DTI analysis, microstructural damages in the white mater fibers of the corpus callosum and to assess its association with long‐term disability in a large prospective cohort of SuS patients.

## METHODS

### Study population

All patients participated in the national multicenter CarESS (Phenotypic and Etiological Characterization of Susac Syndrome—National Clinical Research Hospital Program) study. CarESS study is an ongoing cohort study that started in December 2011 and included all consecutive case of patients with SuS referred to the French reference center (Department of Internal Medicine, Bichat Claude Bernard Hospital, Paris). Inclusion criteria were a minimal age of 18 years old and Susac syndrome defined either by (i) the triad of encephalopathy with typical brain MRI abnormalities, cochleo‐vestibular damage including unilateral or bilateral sensorineural hearing loss on the audiometry and multiple occlusions of retinal central artery branches and/or retinal vasculitis on retinal fluorescein angiography or (ii) at least two of the three aforementioned criteria without any alternative diagnosis, as required by current diagnostic criteria [4].

### Data collection

Age at diagnosis, gender, physical examination, fundoscopy, retinal angiography, visual acuity, visual field, audiometry, cerebrospinal fluid (CSF), brain MRI, and treatment data were systematically collected. The CarESS study was designed with a follow‐up including fundoscopy, automated visual field testing, audiometry, and brain MRI at 1, 3, 6, 12 months after diagnosis, and then annually for 5 years and/or in the case of a relapse. A relapse was defined by new clinical symptoms or signs and new abnormalities on retinal angiography, audiometry, or brain MRI leading to treatment intensification. Data were collected prospectively and analyzed retrospectively.

### MRI acquisition and processing

MRI scans were obtained with a 3‐T MRI scanner performed at Bichat hospital (GE Medical Systems Discovery MR750). The MRI protocol included 2D‐DTI sequences, 15 directions, slice thickness 4 mm, and morphological sequences not used in the present study. All MRI were processed in Olea Sphere 3.0 SP 26 (Olea Medical, La Ciotat, France), generating 3D tractography maps and parametrical FA and MD maps. MD and FA were analyzed through DTI in fibers (i.e., tracts) crossing three regions of interest (ROIs): the corpus callosum as a whole, the genu, and the splenium of the corpus callosum. Two experienced neuroradiologists (AG and MCHF) blinded from clinical data independently read all MRI.

### Primary outcome

The primary outcome was the resumption of work (RW) at last follow‐up. Failure to return to work was considered a proxy for disability.

### Standard protocol approvals, registrations, and patient consents

The CarESS study has been approved by the Committee for the Protection of Persons (CPP Ile de France 1, Paris, France; IRB00008522). All patients signed informed consent. Detailed inclusion and exclusion criteria for the CarESS study are available on the ClinicalTrials.gov portal (Identifier: NCT01481662).

### Data availability statement

Anonymized data not published within this article are available from the corresponding author upon reasonable request.

### Statistical analysis

Continuous variables are expressed as median [first quartile (Q1)–third quartile (Q3)] or as mean (standard deviation). Categorical variables are expressed as numbers and percentages. Data were compared using chi‐squared test (or Fisher) for dichotomous variables and Mann–Whitney test for continuous variables. The Pearson rank correlation test was used to determine correlations between variables, with r being the Pearson correlation coefficient. Relationships between temporal brain microstructural changes and RWA were estimated using simple and multiple linear regression. All tests were adjusted on age, gender, number of lesions in the white matter on standard T2/FLAIR sequences on first MRI, immunosuppressive therapy, and relapses. A *p*‐value of less than 0.05 was considered significant. The Bonferroni test was used for multiple‐comparison correction. Analyses were performed in R (https://www.r‐project.org).

## RESULTS

### Population characteristics

Fifty‐seven patients from eight different centers in France with SuS were enrolled in the CarESS (ClinicalTrials.gov Identifier: NCT01481662) study. Among them, 22 patients included at the principal investigator's center who had at least two brain MRI with DTI performed at diagnosis and during follow‐up were analyzed for the primary outcome. Sixteen patients (73%) were women and the median (IQR) age at diagnosis was 36 (25;42) years. The triad (i.e., brain, eye and ear involvement) was complete in 95% (*n* = 21/22) of patients, either at disease onset, or during disease course. CNS was affected in all patients. All but one patients received steroids alone or in combination with IS agents (*n* = 11) and/or IVIg (*n* = 7). Antiplatelet therapy was given in all but one patients (95%). Fifteen (*n* = 15/22, 68.2%) patients relapsed at least once during follow‐up with a median number of 1 (0;2) relapse per patient (Table 1).

**TABLE 1:** Demographic, clinical characteristics at diagnosis and treatment received in SuS patients.

	All, *n* = 22	No RW, *n* = 7	RW, *n* = 15	RR (95% CI)
Age, years	36 (25;42)	50 (31;46)	35 (24;42)	
Female gender, *n* (%)	16 (73)	6 (86)	10 (67)	2.2 (0.5–13.4)
Triad at onset, *n* (%)	20 (91)	7 (100)	13 (87)	—
Neurologic signs, *n* (%)	22 (100)	7 (100)	15 (100)	—
Headache	17 (77)	6 (86)	11 (73)	1.8 (0.4–10.5)
Encephalopathy	13 (59)	5 (71)	8 (53)	1.7 (0.5–6.9)
Behavioral, conduct or mood disorder	12 (54)	4 (57)	8 (67)	1.1 (0.35–3.8)
Motor impairment	7 (32)	3 (43)	4 (27)	1.6 (0.5–4.9)
Ophthalmic signs, *n* (%)	13 (5)	7 (100)	13 (87)	—
Visual field loss	6 (27)	2 (29)	4 (27)	1.1 (0.27–3.41)
Visual acuity loss	4 (18)	4 (57)	0 (0)	6.0 (2.1–16.8)
Arterial occlusion	18 (82)	6 (86)	12 (80)	1.3 (0.3–7.9)
Hyperfluorescence	8 (36)	1 (14)	7 (47)	0.3 (0.05–1.4)
Cochleo‐vestibular signs, *n* (%)	15 (68)	7 (100)	15 (100)	—
Tinnitus	5 (23)	4 (57)	5 (33)	1.9 (0.6–6.4)
Vertigo	8 (36)	2 (29)	6 (40)	0.7 (0.2–2.4)
Ataxia	6 (27)	2 (29)	4 (27)	1.1 (0.3–3.4)
Hearing loss, >40 db	11 (50)	4 (57)	7 (47)	1.3 (0.4–4.5)
CSF				
Proteins >0.4 g/L, *n* (%)	21 (95)	6 (86)	15 (100)	0.3 (0.1–1.5)
Proteins, g/L	1.1 (0.9;1.8)	1.2 (1.1;2.3)	1 (0.6;1.6)	—
Treatment ever received, *n* (%)				
Corticosteroid	21 (95)	7 (100)	14 (93)	—
IS drugs	11 (50)	6 (86)	5 (33)	6 (1.2–35.3)
IVIG	7 (32)	2 (29)	5 (33)	0.9 (0.2–2.8)
Antiplatelet therapy	21 (95)	7 (100)	14 (93)	—
Follow‐up				
Duration, years	6 (5;8)	8 (3.5;9)	6 (5;7.5)	—
Relapse per patient, *n*	1 (0;2)	2 (1;4)	1 (0;1)	—
MRI per patient, [Q1;Q3]	5.6 [4.2; 7]	6.4 [3.5; 9]	5.2 [4.5; 6.5]	—

*Note*: Continuous variables are given as median (first quartile; third quartile) or as mean (standard deviation).

Abbreviations: CI, confidence interval; CSF, cerebrospinal fluid; db, decibels; IS, immunosuppressive; IVIG, intravenous immunoglobulin; RR, relative risk; RW, resumption of work; SuS, Susac syndrome.

### Brain microstructural changes through DTI parameters

**FIGURE 1:**
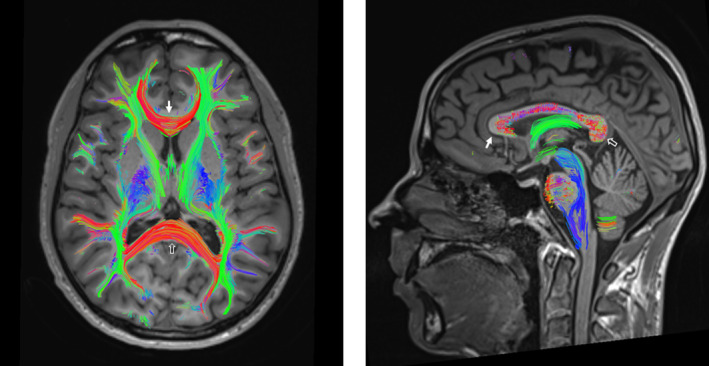
Brain DTI analysis. Tractography overlaid on a 3D GE T1‐weighted MRI of a normal subject, showing fibers in the brain. Due to their right–left orientation, fibers crossing the corpus callosum appear in red. The genu and splenium of the corpus callosum are indicated with solid white and hollow arrows, respectively.

One hundred and twenty three DTI‐MRI—accounting for a mean of 6.8 [4.25;7] MRI per patient—were post‐processed for fractional anisotropy (FA) and mean diffusivity (MD) analysis. FA and MD were analyzed in tracts crossing three regions of interest (ROIs): the corpus callosum as a whole, the genu of the corpus callosum, and the splenium of the corpus callosum (Figure 1). Interobserver agreement was evaluated on each patient first MRI and estimated by intra‐class correlation (ICC). Interobserver agreement was excellent for MD and FA in tracts (ICC = 0.997) and ROIs (ICC = 0.967), and particularly regarding the tract of corpus callosum (for FA, ICC = 0.998; for MD, ICC = 0.999).

### Outcome

Over a median (IQR) follow‐up of 6 [5; 8] years, 15 (68%) patients returned to work: 11 full‐time, including 9 at their original post, and 4 part‐time. Univariate analysis showed that failure of resumption of work (RW) was associated with a visual acuity loss at diagnosis (RR (95% CI): 6 (2.1–16.8); *p* = 0.005), a higher number of relapses (*n* = 2 (1;4) vs. *n* = 1 (0;1); *p* = 0.011) and—though not statistically significant—the use of IS drugs (RR (95% CI): 6 (1.2–35.3), *p* = 0.063) (Table 1). In addition, failure to return to work tended to be associated with a higher level of disability assessed at the final follow‐up (Table S1).

**FIGURE 2:**
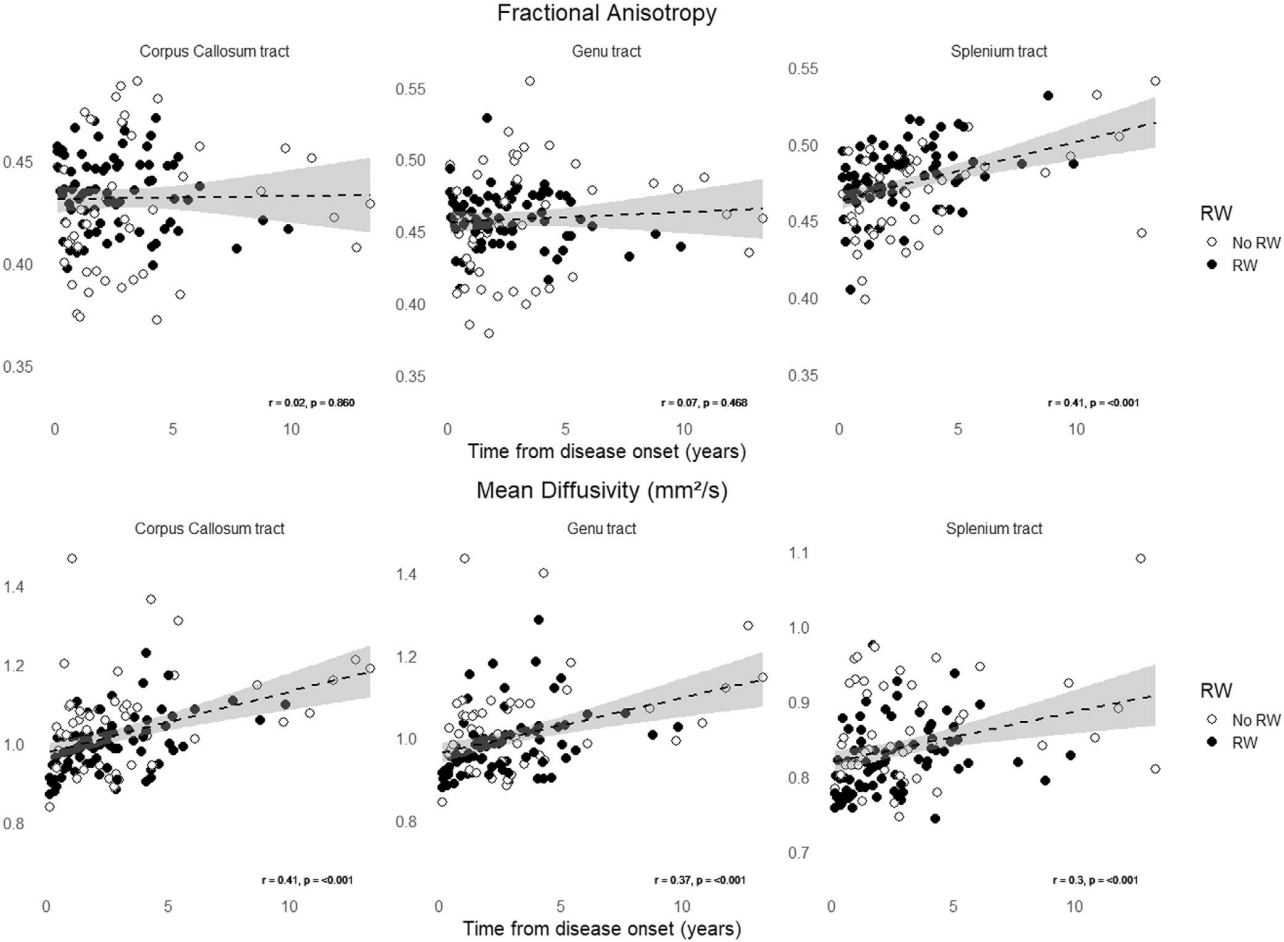
Analysis of fractional anisotropy and mean diffusivity on brain DTI during follow‐up. Overtime analysis of the fractional anisotropy (FA, upper row) and the mean diffusivity (MD, lower row) in the corpus callosum (CC) as a whole, the genu, and the splenium in SuS patients according to the resumption of work (RW) status at last follow‐up. The Pearson rank correlation test was used to determine correlations between variables, with r being the Pearson correlation coefficient.

Analysis of the first brain DTI‐MRI, performed a median of 6.2 [3.9; 15.0] months after the first manifestation of SuS, showed no statistically significant difference in FA and MD in any tracts crossing the corpus callosum according to work status at the last follow‐up (Table 1). By analyzing brain DTI IRM as a whole (i.e., per group), we observed that mean FA and MD were respectively lower and higher in patients who did not return to work in any of the ROIs involving the corpus callosum (Table 2). The association between microstructural alterations in fibers crossing the corpus callosum and work status remained significant after adjustment on age, gender, number of white matter lesions on standard T2/FLAIR sequences, IS drugs, and relapses (Table 2). Of note, the number of MRI scans performed did not differ according to the RW status (mean number of MRI per patient of 6.4 [3.5;9] vs. 5.2 [4.5;6.5], *p* = 0.100). Eventually, longitudinal analysis of the brain DTI IRM per patient revealed that MD significantly increased in the three ROIs overtime but without association with work status (Figure 2).

**TABLE 2:** Fractional anisotropy and mean diffusivity analysis in SuS patients.

Tracts	First DTI‐MRI			Whole DTI‐MRI^b^		
	No RW, *n* = 7	RW, *n* = 15	*p*‐value^a^	No RW, *n* = 7	RW, *n* = 15	*p*‐value^c^
FA						
Corpus callosum	0.44 (0.05)	0.44 (0.03)	0.928	0.42 (0.05)	0.44 (0.03)	<0.001
Genu	0.48 (0.08)	0.46 (0.02)	0.999	0.45 (0.07)	0.46 (0.02)	<0.001
Splenium	0.47 (0.06)	0.47 (0.01)	0.999	0.47 (0.04)	0.48 (0.02)	<0.001
MD, mm^2^/s						
Corpus callosum	1.04 (0.22)	0.95 (0.1)	0.999	1.06 (0.14)	0.99 (0.08)	<0.001
Genu	1.02 (0.14)	0.92 (0.09)	0.640	1.02 (0.13)	0.96 (0.08)	<0.001
Splenium	0.84 (0.11)	0.80 (0.05)	0.232	0.84 (0.11)	0.82 (0.06)	<0.001

*Note*: First DTI‐MRI performed 6.2 [3.9;15.0] months after disease onset. The Bonferroni test was used for multiple comparison correction.

Abbreviations: FA, fractional anisotropy; MD, mean diffusivity; RW, resumption of work.

^a^ Comparison performed after adjustment on age and number of lesions in the white matter on standard T2/FLAIR sequences.

^b^ Analysis per group.

^c^ Comparison performed after adjustment age, gender, number of lesions in the white matter on standard T2/FLAIR sequences on first MRI, IS drugs, and relapses.

## DISCUSSION

In 2011, a national clinical based cohort (CarESS study) was built in order to better characterize the epidemiological, clinical and etiological features of SuS. In this study, all new patients prospectively followed at the principal investigator's center and for whom brain diffusion tensor imaging (DTI) were available were included. We show that microstructural damage in fibers crossing the corpus callosum is associated with the inability to resume work in SuS.

A previous study investigating DTI in nine SuS patients first identified microstructural impairment in the corpus callosum [17]. Such microstructural lesions of corpus callosum were confirmed in a more recent cross‐sectional study involving seven patients [15]. In our study, we observed an association between microstructural damages assessed by mean diffusivity (MD) and fractional anisotropy (FA) and poor outcome. In SuS, neurocognitive evaluation performed while SuS was in long lasting remission revealed that 40% of patients had impaired recall and executive functions [7]. Our findings are consistent with studies showing an association between altered structural integrity of the white matter and cognitive impairments in cerebral small vessel diseases [14, 18].

Treatment of SuS is empirical, based on the hypothesis of an autoimmune endotheliopathy supporting the use of immunosuppressive (IS) drugs in addition to corticosteroids. In the absence of randomized trials, it remains unclear how much and for how long treatment is required and what is the real impact of IS drugs on the natural history of SuS. Because brain microstructural damage in the corpus callosum occurs early and is associated with poor outcome, brain DTI might help to identify SuS patients with a more severe disease in whom the use of IS drugs might be required at early stage.

Surprisingly, we observed a dissociation between MD and FA in the splenium of the corpus callosum, with a significant increase in FA during follow‐up. Kleffner et al. found no decrease in FA in the splenium of nine SuS patients, whereas the genu of the corpus callosum was affected [17]. Several hypotheses could explain such an unexpected finding. The normal splenium appears to exhibit a distinct DTI behavior, with increased FA as compared to the rest of the corpus callosum [19]. Lesional mechanisms in the splenium might also be specific. For instance, cytotoxic lesions of the corpus callosum, primarily affecting the splenium, could be due to intramyelinic edema, which might partially explain the reversibility of these lesions [20, 21]. In traumatic brain injury, splenium lesions are also observed, but these are likely caused by diffuse axonal injury leading to axonal degeneration [22]. In Susac syndrome, the lesions are probably due to direct microvascular impairment. The combined effects of the splenium's microstructural specificity and the lesions caused by Susac syndrome might partially explain the dissociation observed between MD and FA. Another hypothesis could involve other causes of increased FA, such as tighter axonal fibers or changes in the perivascular composition, with an increase in the collagenous component [19].

Our study has several strengths as it is the first study with a protracted follow‐up analyzing DTI in a prospective cohort of SuS patients starting at the acute symptomatic phase. Comprehensive clinical, audiogram, and retinal angiography data were systematically assessed. DTI‐MRI scans were performed using a standardized protocol.

Our study also has several limitations. First, the conclusions are based on a limited number of patients and our study did not include any age‐ or sex‐matched control group. Secondly, DTI analysis was not performed on normal‐appearing white matter, to the exclusion of macroscopic lesions, in order to assess whether lesion‐independent microstructural damage also correlates with clinical outcomes. We used a tract‐based approach and DTI parameters were collected for the entire tract and not for the traced ROI itself. We were thus unable to exclude T2‐FLAIR focal lesions from these ROIs, as this would have compromised the integrity of the entire selected tract. However, we previously failed to demonstrate any association between brain lesion burden assessed by brain MRI and disability in SuS [7]. In addition, we did not observed any correlation between the number white matter lesions observed on standard T2/FLAIR sequences and the degree of microstructural damage (Figure S1). Third, there is no validated damage index score in SuS and other factors than disease‐related disability may impede the return to work such as age, socioeconomic status, previous sick leave and unemployment, or depression. Fourth, patients who did not resume work might have a more severe disease at onset, as suggested by a higher proportion of patients treated with IS drugs and a higher frequency of visual defects, knowing that the model was adjusted for potential confounders. Fifth, despite a careful design of the CarESS study, not all patients have been included because of missing data, leading to selection bias. Sixth, DTI analysis might be impeded by technical issues including differences in image acquisition parameters between 1.5T and 3T scans, numbers of gradient directions for parameter acquisition, and methods for post‐processing analysis [23, 24].

In conclusion, microstructural damages involving the corpus callosum evaluated by DTI‐MRI is associated with poor outcome in SuS.

## AUTHOR CONTRIBUTIONS

**Augustin Gaudemer:** Conceptualization; investigation; methodology; formal analysis; writing – original draft; writing – review and editing. **Marie‐Cécile Henry‐Feugeas:** Investigation; formal analysis; writing – review and editing. **Marion Peyre:** Investigation; writing – review and editing. **Alexandra Kachaner:** Investigation; writing – review and editing. **Isabelle Klein:** Conceptualization; methodology; investigation; writing – review and editing. **Antoine Khalil:** Investigation; writing – review and editing. **Thomas Papo:** Conceptualization; supervision; methodology; investigation; writing – review and editing; funding acquisition. **Karim Sacré:** Conceptualization; supervision; methodology; investigation; formal analysis; funding acquisition; writing – original draft; writing – review and editing.

## FUNDING INFORMATION

This work was supported by grants from the French Ministry of Health (PHRC 2005‐090039; CRC 2019‐19059), the University Paris Cité, and the Assistance Publique Hôpitaux de Paris.

## CONFLICT OF INTEREST STATEMENT

No conflicts of interest related to this article.

## Supporting information


Data S1.

## Data Availability

The data that support the findings of this study are available from the corresponding author (KS), upon reasonable request. This has already been assessed in the ‘Methods’.

## Appendix

List of collaborators (French Susac Study Group) to be added in PubMed: Olivier Aumaitre, Anne Catherine Bachoud Levi, Naima Beldjoudi, Marie Bodenant, Jean Capron, Marie‐Paule Chauveheid, Fleur Cohen Aubart, Clémence David, Thomas de Brouker, Solene De Gaalon, Sabrina Debruxelles, Serge Doan, Antoine Dossier, Emmanuel Ellie, Chrystelle François, Sophie Godard‐Ducceschi, Bertrand Godeau, Tiphaine Goulenok, Deborah Grosset‐Janin, Caroline Halimi, Mohamed Hamidou, Eric Jouvent, Cedric Laouenan, Bertand Lapergue, Alain Le Quellec, Arthur Mageau, Elisabeth Medeiros de Bustos, Shirine Mohamed, Luc Mouthon, Jean‐Baptiste Noury, Diane Rouzaud, Mélanie Roriz, Guillaume Turc, and Anne Wacongne.

## References

[1] Dörr J , Krautwald S , Wildemann B , et al. Characteristics of Susac syndrome: a review of all reported cases. Nat Rev Neurol. 2013;9(6):307‐316. doi:10.1038/nrneurol.2013.82 23628737

[2] David C , Sacré K , Henri‐Feugeas MC , et al. Susac syndrome: a scoping review. Autoimmun Rev. 2022;21(6):103097. doi:10.1016/j.autrev.2022.103097 35413469

[3] Gross CC , Meyer C , Bhatia U , et al. CD8+ T cell‐mediated endotheliopathy is a targetable mechanism of neuro‐inflammation in Susac syndrome. Nat Commun. 2019;10(1):5779. doi:10.1038/s41467-019-13593-5 31852955 PMC6920411

[4] Kleffner I , Dörr J , Ringelstein M , et al. Diagnostic criteria for Susac syndrome. J Neurol Neurosurg Psychiatry. 2016;87(12):1287‐1295. doi:10.1136/jnnp-2016-314295 28103199

[5] Machado S , Jouvent E , Klein I , et al. Cognitive dysfunction and brain atrophy in Susac syndrome. J Neurol. 2020;267(4):994‐1003. doi:10.1007/s00415-019-09664-8 31828475

[6] Peyre M , Mageau A , Henry Feugeas MC , et al. Risk factors for severe hearing loss in Susac syndrome: a national cohort study. Eur J Neurol. 2024;31(5):e16211. doi:10.1111/ene.16211 38235955 PMC11235986

[7] Scheifer C , Henry Feugeas MC , Roriz M , et al. Brain magnetic resonance imaging lesion load at diagnosis, severity at onset and outcomes in Susac syndrome: a prospective cohort study. Eur J Neurol. 2021;12:121‐129. doi:10.1111/ene.15062 34382290

[8] Gons RAR , de Laat KF , van Norden AGW , et al. Hypertension and cerebral diffusion tensor imaging in small vessel disease. Stroke. 2010;41(12):2801‐2806. doi:10.1161/STROKEAHA.110.597237 21030696

[9] Maillard P , Seshadri S , Beiser A , et al. Effects of systolic blood pressure on white‐matter integrity in young adults in the Framingham heart study: a cross‐sectional study. Lancet Neurol. 2012;11(12):1039‐1047. doi:10.1016/S1474-4422(12)70241-7 23122892 PMC3510663

[10] de Laat KF , van Norden AGW , Gons RAR , et al. Diffusion tensor imaging and gait in elderly persons with cerebral small vessel disease. Stroke. 2011;42(2):373‐379. doi:10.1161/STROKEAHA.110.596502 21193751

[11] van Norden AGW , de Laat KF , van Dijk EJ , et al. Diffusion tensor imaging and cognition in cerebral small vessel disease: the RUN DMC study. Biochim Biophys Acta. 2012;1822(3):401‐407. doi:10.1016/j.bbadis.2011.04.008 21549191

[12] Tuladhar AM , Tay J , van Leijsen E , et al. Structural network changes in cerebral small vessel disease. J Neurol Neurosurg Psychiatry. 2020;91(2):196‐203. doi:10.1136/jnnp-2019-321767 31744851

[13] Hollocks MJ , Lawrence AJ , Brookes RL , et al. Differential relationships between apathy and depression with white matter microstructural changes and functional outcomes. Brain J Neurol. 2015;138(Pt 12):3803‐3815. doi:10.1093/brain/awv304 PMC4655344 26490330

[14] Li X , Ma C , Sun X , et al. Disrupted white matter structure underlies cognitive deficit in hypertensive patients. Eur Radiol. 2016;26(9):2899‐2907. doi:10.1007/s00330-015-4116-2 26615558

[15] Johnson P , Chan J , Vavasour I , et al. Quantitative MRI findings indicate diffuse white matter damage in Susac syndrome. Mult Scler J ‐ Exp Transl Clin. 2022;8(1):205521732210788. doi:10.1177/20552173221078834 PMC8851927 35186315

[16] Kleffner I , Deppe M , Mohammadi S , et al. Diffusion tensor imaging demonstrates fiber impairment in Susac's syndrome. J Neurol Sci. 2009;283(1–2):254. doi:10.1016/j.jns.2009.02.062 17959768

[17] Kleffner I , Deppe M , Mohammadi S , et al. Neuroimaging in Susac's syndrome: focus on DTI. J Neurol Sci. 2010;299(1–2):92‐96. doi:10.1016/j.jns.2010.08.028 20850137

[18] da Silva PHR , Paschoal AM , Secchinatto KF , et al. Contrast agent‐free state‐of‐the‐art magnetic resonance imaging on cerebral small vessel disease—part 2: diffusion tensor imaging and functional magnetic resonance imaging. NMR Biomed. 2022;35(8):e4743. doi:10.1002/nbm.4743 35429070

[19] Chepuri NB , Yen YF , Burdette JH , Li H , Moody DM , Maldjian JA . Diffusion anisotropy in the corpus callosum. AJNR Am J Neuroradiol. 2002;23(5):803‐808. 12006281 PMC7974733

[20] Tetsuka S . Reversible lesion in the splenium of the corpus callosum. Brain Behav. 2019;9(11):e01440. doi:10.1002/brb3.1440 31588684 PMC6851813

[21] Starkey J , Kobayashi N , Numaguchi Y , Moritani T . Cytotoxic lesions of the corpus callosum that show restricted diffusion: mechanisms, causes, and manifestations. Radiographics. 2017;37(2):562‐576. doi:10.1148/rg.2017160085 28165876

[22] Venkatasubramanian PN , Keni P , Gastfield R , et al. Diffusion tensor imaging detects acute and subacute changes in corpus callosum in blast‐induced traumatic brain injury. ASN Neuro. 2020;12:1759091420922929. doi:10.1177/1759091420922929 32403948 PMC7238783

[23] van den Brink H , Doubal FN , Duering M . Advanced MRI in cerebral small vessel disease. Int J Stroke. 2023;18(1):28‐35. doi:10.1177/17474930221091879 35311609 PMC9806457

[24] Soares JM , Marques P , Alves V , Sousa N . A hitchhiker's guide to diffusion tensor imaging. Front Neurosci. 2013;7:31. doi:10.3389/fnins.2013.00031 23486659 PMC3594764

