# Supplementary material for: Brain microstructural damage through serial diffusion tensor imaging and outcomes in Susac syndrome: A prospective cohort study
Source: Eur J Neurol. 2024 Dec 16;32(1):e70002. doi: 10.1111/ene.70002 (PMC11649952; doi:10.1111/ene.70002)
Supplement: Supplementary file 1 — Data S1. [file ENE-32-e70002-s001.docx]

**SUPPLEMENTARY MATERIAL**

**Table S** **Disabilities in SuS patients at last follow up**

|  | missing | No RW n=7 | RW  n=15 | p |
| --- | --- | --- | --- | --- |
| Motor impairment | 0 (0) | 2 (29) | 0 (0) | 0.091 |
| Visual acuity loss | 0 (0) | 1 (14) | 1 (7) | 0.999 |
| Hearing loss >70 db or need for hearing aids | 0 (0) | 7 (100) | 9 (60) | 0.120 |
| MoCA score <27 | 5 (23) | 5 (71) | 5 (33) | 0.172 |
| IADL <8 | 0 (0) | 0 (0) | 0 (0) | 0.999 |

The level of disability was assessed at the last follow-up (median (IQR) of 6 [5; 8] years) expect for neurocognitive assessment using the MoCA score, which was performed 20.3 (14.4-41.8) months after diagnosis, while SuS was in remission with no subsequent relapse.

Autonomy was tested with the Lawton Instrumental Activities of Daily Living (IADL) scale.

Variables are expressed as numbers and percentages. Data were compared using Fisher test.

**Figure S1** **White matter lesions on standard T2/FLAIR sequences and microstructural damage**


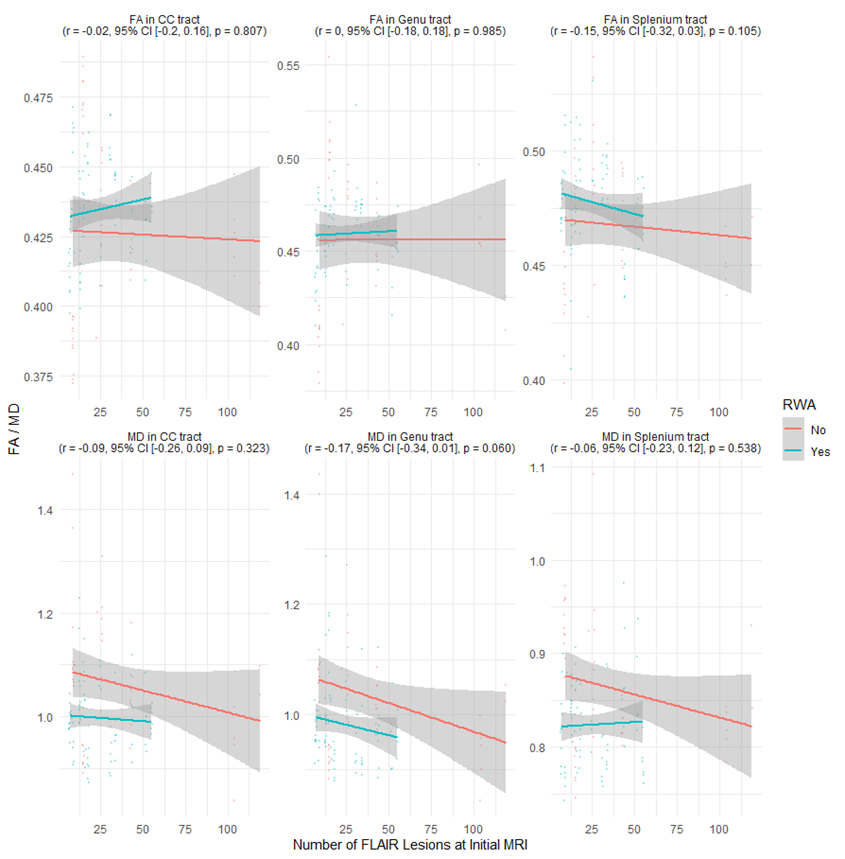


Analysis of the fractional anisotropy (FA, upper row) and the mean diffusivity (MD, lower row) in the corpus callosum (CC) as a whole, the genu and the splenium in SuS patients according to the number of T2/FLAIR lesions in performed on the on first MRI. The Pearson rank correlation test was used to determine correlations between variables, with r being the Pearson correlation coefficient.
